# Outcomes of Surgical Repair of Incisional Hernia in Patients With Severe and Morbid Obesity: A Comparative Study

**DOI:** 10.7759/cureus.55782

**Published:** 2024-03-08

**Authors:** Islam Omar, Amr Anany, Mohamed Ismaiel, Abby Townsend, Jeremy Wilson, Conor Magee

**Affiliations:** 1 Department of General Surgery, The Hillingdon Hospitals National Health Service (NHS) Foundation Trust, Uxbridge, GBR; 2 Department of General Surgery, Charing Cross Hospital, Imperial College National Health Service (NHS) Trust, London, GBR; 3 Department of General Surgery, Altnagelvin Hospital, Londonderry, GBR; 4 Department of General Surgery, Wirral University Teaching Hospital National Health Service (NHS) Foundation Trust, Wirral, GBR

**Keywords:** safety, outcomes, ventral hernia, morbid obesity, incisional hernia

## Abstract

Introduction

Incisional hernia (IH) is a common complication after open and minimal access abdominal surgery. The current practice guidelines recommend weight reduction to achieve a body mass index (BMI) < 35 kg/m^2^ before surgical repair of ventral hernias. However, this could be challenging to achieve, especially in emergency presentations. This study aims to assess the safety of surgical repair of IH in patients with BMI ≥35 kg/m^2^.

Methods

A retrospective comparative study has been conducted to include all patients who had surgical repair of IH on an elective and emergency basis in a UK District General Hospital. The patients were divided into two groups. Group I BMI < 35 kg/m^2^ and Group II with BMI ≥35 kg/m^2^. A comparison was made between the two groups according to demographics, comorbidities, hernia characteristics, operative data, and outcomes.

Results

The study included 239 patients, 181 in Group I and 58 in Group II. Morbid obesity was associated with male patients, and they were younger than Group I, p= 0.001 and 0.013, respectively. 13.8% of Group I had DM compared to 29.3% in Group II, p= 0.007. There were no significant differences in hernia characteristics or mode of surgery between the two groups. However, Group II had more overall and wound-related complications, p= <0.001 each. There were no significant differences in 30-day and 90-day mortality, recurrence rate, or 90-day readmissions.

Conclusions

Surgical repair of IH in patients with severe and morbid obesity is associated with more overall and wound-related complications.

## Introduction

Incisional hernias (IH) in patients with morbid obesity represent a real challenge and create a vicious cycle that is hard to break. On the one hand, obesity is a known predisposing factor for developing IH after abdominal procedures [1]. On the other hand, large IH could limit physical activities and lead to social isolation due to disfigurement, exacerbating obesity [2].

The surgical repair of IH is associated with symptom relief and improved quality of life [3]. However, in the presence of obesity, surgical repair could be associated with an increased risk of complications and recurrence [4,5]. The current practice guidelines recommend weight reduction to achieve a body mass index (BMI) < 35 kg/m2 before surgical repair of ventral hernias [6]. However, this could be challenging to achieve in IH with morbid obesity, given the long waiting lists, the persistence of obesity that can lead to more complex cases and the presentation of incarceration on an emergency basis [7]. This study aims to assess the safety of surgical repair of IH in patients with BMI ≥35 kg/m2.

## Materials and methods

Study design

A comparative retrospective cohort single-centre study from 2013 to 2021.

Setting

High volume acute service district general hospital in the United Kingdom.

Participants

Inclusion Criteria

Study participants included all patients who had surgical repair of incisional hernia on an elective and emergency basis and had complete data.

Variables examined

Patients' Demographics, Comorbidities, and Risk Factors

The primary outcome parameters included all-cause and wound-related complications and mortality rates. The secondary outcome parameters included length of hospital stay, need for high-level care, unplanned 90-day readmissions, and recurrence rate. The patients were divided into two groups; Group I had BMI <35 and Group II had ≥35. A comparison was made between the two groups according to the different variables examined.

Data source/bias

Data was collected from computerised records and patient case notes.

Study size

The total number of patients admitted for surgical repair of incisional hernias with complete data.

Statistical analysis

Data were analysed using the statistical software package Statistical Package for Social Sciences (SPSS), version 20.0 (IBM Corp., Armonk, NY). The Kolmogorov-Smirnov test was used to verify the normality of distribution. Continuous data were described using range (minimum and maximum), mean, standard deviation, and median. Comparisons between groups for categorical variables were assessed using the Chi-square test (Fisher or Monte Carlo). Student t-test was used to compare two groups for normally distributed quantitative variables. Mann-Whitney test was used to compare two groups for non-parametric data. Spearman coefficient was used to assess correlation. 

## Results

A total of 296 patients who had operations for IH were identified; 57 of them had incomplete data and were consequently excluded from the study. The analysis included 239 patients, 181 with BMI <35 (Group I) and 58 patients with BMI≥ 35 (Group II). 

In Group I, 45 had emergency repair and 136 had elective repair. In Group II, 28 had emergency repair and 30 had elective repair. Moreover, in regards to gender, in Group II, 44 (75.9%) were females, compared to 94 (51.9%) in Group I, p=0.001. Group II patients were significantly younger, p= 0.013.

The comparison of the identified comorbidities showed a significant difference with diabetes mellitus (DM) only, where Group II had more patients, 17 (29.3%) with DM, compared to 25 (13.8%) in Group I, p = 0.007. Additionally, Group I included more patients with malignancy compared to Group II, p= p= 0.013. The comparison of the other comorbidities and risk factors did not show significant differences between the two groups (Table 1).

**Table 1:** Relation between BMI and demographics, comorbidities, and other risk factors (n=239) DM: Diabetes mellitus, COPD: Chronic obstructive pulmonary disease, CKD: Chronic kidney disease, SD: Standard deviation, x^2^: Chi-square test, FE: Fisher Exact, U: Mann Whitney test, t: Student t-test, p: p-value for association between the studied categories *: Statistically significant at p ≤ 0.05

Variables	BMI (kg/m^2^)		Test of sig.	p
	Group I <35	Group II ≥35		
Gender	(n = 181)	(n = 58)		
Male	87 (48.1%)	14 (24.1%)	X^2^=10.307^*^	p= 0.001^*^
Female	94 (51.9%)	44 (75.9%)		
Age (years)	(n = 181)	(n = 58)		
Median (Min-Max)	64 (26 – 90)	59.5 (28– 82)	t=2.490^*^	p= 0.013^*^
Mean ± SD	62.78 ± 14.21	57.53 ± 13.13		
Any comorbidity	(n = 181)	(n = 58)		
No	68 (37.6%)	25 (43.1%)	X^2^=0.566	p= 0.452
Yes	113 (62.4%)	33 (56.9%)		
DM	(n = 181)	(n = 58)		
No	156 (86.2%)	41 (70.7%)	X^2^=7.284^*^	p= 0.007^*^
Yes	25 (13.8%)	17 (29.3%)		
Malignancy	(n = 181)	(n = 58)		
No	134 (74%)	52 (89.7%)	X^2^=6.211^*^	p= 0.013^*^
Yes	47 (26%)	6 (10.3%)		
Connective tissue disease	(n = 181)	(n = 58)		
No	172 (95%)	57 (98.3%)	X^2^=1.156	^FE^p= 0.458
Yes	9 (5%)	1 (1.7%)		
COPD	(n = 181)	(n = 58)		
No	163 (90.1%)	52 (89.7%)	X^2^=0.008	p= 0.930
Yes	18 (9.9%)	6 (10.3%)		
CKD	(n = 181)	(n = 58)		
No	167 (92.3%)	53 (91.4%)	X^2^=0.047	^FE^p= 0.785
Yes	14 (7.7%)	5 (8.6%)		
Other risk factors				
Chemotherapy	(n =181)	(n =58)		
No	172 (95.0%)	57 (98.3%)	X^2^= 1.156	^FE^p= 0.458
Yes	9 (5.0%)	1 (1.7%)		
Radiotherapy	(n =181)	(n =58)		
No	178 (98.3%)	58 (100%)	X^2^= 0.974	^FE^p= 1.000
Yes	3 (1.7%)	0 (0%)		

Table 2 shows the comparison according to the hernia characteristics, operative details, and outcome parameters. There was a wide variation in the hernial defect size; these ranged from 1.5 by 1.5 cm to 20 by 14 cm. Additionally, there were no significant differences in hernia characteristics or mode of surgery between the two groups.

**Table 2:** Relation between BMI and hernia characteristics, operative details, and outcome parameters (n=239) CD: Clavien-Dindo, HDU: High dependency unit, SD: Standard deviation, x^2^: Chi-square test, FE: Fisher Exact, U: Mann Whitney test, MC: Monte Carlo, p: p-value for association between the studied categories *: Statistically significant at p ≤ 0.05

Variables	BMI (kg/m^2^)		Test of sig.	p
	Group I <35	Group II ≥35		
Multiple defects	(n = 176)	(n = 58)		
No	114 (64.8%)	44 (75.9%)	X^2^=2.446	p= 0.118
Yes	62 (35.2%)	14 (24.1%)		
Primary/recurrent	(n = 178)	(n = 57)		
Primary	132 (74.2%)	41 (71.9%)	X^2^=0.110	p= 0.740
Recurrent	46 (25.8%)	16 (28.1%)		
Stoma on presentation	(n =179)	(n =57)		
No	165 (92.2%)	56 (98.2%)	X^2^= 2.674	^FE^p= 0.127
Yes	14 (7.8%)	1 (1.8%)		
Mode	(n =177)	(n =557)		
Lap	31 (17.5%)	7 (12.3%)	X^2^= 1.861	^MC^p= 0.328
Open	145 (81.9%)	49 (86%)		
Assisted	1 (0.6%)	1 (1.8%)		
Adhesiolysis	(n = 178)	(n = 57)		
No	86 (48.3%)	32 (56.1%)	X^2^=1.058	p= 0.304
Yes	92 (51.7%)	25 (43.9%)		
Duration of surgery (min)	(n = 171)	(n = 55)	U=4327.0	p= 0.373
Median (Min-Max)	100 (11 - 604)	78 (22 - 375)		
Mean ± SD	128.1 ± 105.0	109.53 ± 81.80		
Postop HDU/ICU	(n =179)	(n =58)		
No	160 (89.4%)	47 (81%)	^_X_2^= 2.763	p= 0.096
Yes	19 (10.6%)	11 (19%)		
Hospital stay (days)	(n =181)	(n =58)		
Median (Min. – Max.)	3 (0 – 169)	4 (0 – 57)	U= 4676.0	p= 0.208
Mean ± SD.	6.2 ± 14	7.10 ± 11.1		
Clavien-Dindo	(n =181)	(n =58)		
No CD	111 (61.3%)	22 (37.9%)	^_x_2^= 22.929^*^	^MC^p <0.001^*^
CDI	31 (17.1%)	11 (19%)		
CDII	25 (13.8%)	11 (19%)		
CDIIIA	4 (2.2%)	4 (6.9%)		
CDIIIB	3 (1.7%)	9 (15.5%)		
CD IV	3 (1.7%)	1 (1.7%)		
CDV	4 (2.2%)	0 (0%)		
Wound complications	(n =180)	(n =58)		
No	159 (88.3%)	38 (65.5%)	x^2^= 16.014^*^	^FE^p <0.001^*^
Yes	21 (11.7%)	20 (34.5%)		
Mortality 30 days	(n =180)	(n =58)		
No	176 (97.8%)	58 (100%)	x^2^= 1.311	^FE^p= 0.575
Yes	4 (2.2%)	0 (0%)		
Mortality 90 days	(n =180)	(n =58)		
No	175 (97.2%)	58 (100%)	x^2^= 1.646	^FE^p= 0.339
Yes	5 (2.8%)	0 (0%)		
Recurrence	(n =180)	(n =58)		
No	166 (92.2%)	49 (84.5%)	x^2^= 3.010	p= 0.083
Yes	14 (7.8%)	9 (15.5%)		
90 days readmissions	(n =180)	(n =58)		
No	154 (85.6%)	44 (75.9%)	x^2^= 2.948	p= 0.086
Yes	26 (14.4%)	14 (24.1%)		

A comparison of the outcome parameters showed that Group II had more overall and wound-related complications, p <0.001 each. There were no significant differences in 30-day and 90-day mortality, recurrence rate, or 90-day readmissions.

## Discussion

This study compared the outcomes of the surgical repair of IH between patients with and without morbid obesity. Morbid obesity was associated with overall and wound-related complications.

Obesity and its associated comorbidities increase the risk of local wound complications and lead to a defective healing process and IH after primary abdominal surgery [4,8]. Given the situation's complexity when patients with morbid obesity develop IH, there might be some inclination towards a watchful waiting strategy with this patient group. However, the safety of this approach is questionable [9]. A recent study in the USA showed that obesity was associated with significantly increased odds of nonelective repair of IH [10]. Additionally, nonelective repair of IH was correlated with in-hospital mortality, postoperative complications, and prolonged hospital stay [10].

Another study included 3,908 patients with IH and studied the effect of obesity, diabetes, and smoking on the outcomes [11]. They found that having one or two of these comorbidities significantly increased the risk of surgical site occurrence. Moreover, patients with all three comorbidities had a two-fold increase in odds for all wound morbidity, followed similarly by obese patients with diabetes. They concluded that having multiple comorbidities was associated with the need for interventions for wound complications. This was most evident in patients with all three comorbidities and with obesity and DM.

In this series, 29.3% of the patients with morbid obesity had DM, and 56.9% had at least one medical comorbidity. This could potentially put this group at risk with any surgical intervention. However, the prevalence of malignancy was higher in Group I, 47 (26%), compared to six (10.3%) in Group II. This could be attributed to the age difference between the two groups. The mean age was 62.78 ± 14.21 in Group I and 57.53 ± 13.13 years in Group II, p= 0.013.

Studying the hernia characteristics showed that 62 (35.2%) of Group I and 14 (24.1%) of Group II presented with multiple hernial defects, and 46 (25.8%) of Group I and 16 (28.1%) of Group II had recurrent IH. Ninety-two (51.7%) of Group I and 25 (43.9%) of Group II patients needed adhesiolysis. These findings reflect the complexity of this cohort. Comparing the outcome parameters between the two groups showed a higher complication rate of 62.1% in Group II compared to 38.7% in Group I. This has been demonstrated across all Clavien-Dindo (CD) categories. Additionally, the wound complications in Group II were three-fold (34.5%) compared to Group I (11.7%). However, the 30-day and 90-day mortality rates, hospital stay, the need for high-level care, 90-day readmissions, and recurrence rate did not show significant differences between the two groups.

The available literature presents a blurred picture of the relation between BMI and recurrence rates after ventral and IH repair. Some case series refuted any association between obesity and recurrence [12,13]. On the contrary, other studies reported obesity as an independent risk factor for recurrence [14,15]. A more recent study showed a recurrence rate of 10.5% for BMI> 32 versus 1.7% with BMI ≤32 after IH repair [5]. In our series, the recurrence rate was 14 (7.8%) in Group I and nine (15.5%) in Group II after a mean follow-up of 55.9 SD ± 10.2 (Min - Max; 26.8 - 72.4), with a median of 57.6 months. However, the difference wasn’t statistically significant; p = 0.083.

Despite the safety of IH repair in patients with severe and morbid obesity, our findings highlight the importance of patient selection and optimisation preoperatively to reduce the risk of postoperative complications.

Study strengths and limitations

The limitations of this study included the retrospective nature and relatively small sample size. We endeavoured to mitigate any potential bias that could impact the outcomes. In this respect, we studied and compared the risk factors and comorbidities between the two groups to establish comparability and eliminate confounders. We only excluded patients who did not have complete necessary data that would have made the comparison impossible. Moreover, the data completion and extensive data analysis could present an accurate assessment of this case series. The extended follow-up period is a valuable strength of the study.

## Conclusions

Morbid obesity was associated with the female gender, younger age, and diabetes mellitus. Surgical repair of incisional hernia in patients with morbid obesity is associated with more overall and wound-related complications. There were no significant differences in hospital stay, the need for high-level care, 30-day and 90-day mortality, recurrence rate, or 90-day readmissions.

## References

[1] Scheuerlein H, Settmacher U, Lenschow M, Rauchfuss F (2016). Complex incisional hernias. Arch Clin Gastroenterol.

[2] Dietz UA, Menzel S, Lock J, Wiegering A (2018). The treatment of incisional hernia. Dtsch Arztebl Int.

[3] Manoharan S, Liu G, Crump RT, Karimuddin AA, Scott TM, Sutherland JM (2020). Incisional hernia repair surgery improves patient reported outcomes. Am J Surg.

[4] van Silfhout L, Leenders LA, Heisterkamp J, Ibelings MS (2021). Recurrent incisional hernia repair: surgical outcomes in correlation with body-mass index. Hernia.

[5] Ayik N, Klein P, Grützmann R, Demir R (2019). Long-term Outcome of Incisional Hernia Repairs Using the Erlangen Inlay Onlay Mesh (EIOM) Technique. J Surg Res.

[6] Henriksen NA, Montgomery A, Kaufmann R (2020). Guidelines for treatment of umbilical and epigastric hernias from the European Hernia Society and Americas Hernia Society. Br J Surg.

[7] Campanelli G (2021). Incisional hernia repair: still a complex matter. Hernia.

[8] Walming S, Angenete E, Block M, Bock D, Gessler B, Haglind E (2017). Retrospective review of risk factors for surgical wound dehiscence and incisional hernia. BMC Surg.

[9] Verhelst J, Timmermans L, van de Velde M, Jairam A, Vakalopoulos KA, Jeekel J, Lange JF (2015). Watchful waiting in incisional hernia: is it safe?. Surgery.

[10] Hoffman RD, Danos DM, Lau FH (2021). National health disparities in incisional hernia repair outcomes: an analysis of the Healthcare Cost and Utilization Project National Inpatient Sample (HCUP-NIS) 2012-2014. Surgery.

[11] Alkhatib H, Tastaldi L, Krpata DM (2019). Impact of modifiable comorbidities on 30-day wound morbidity after open incisional hernia repair. Surgery.

[12] Venclauskas L, Jokubauskas M, Zilinskas J, Zviniene K, Kiudelis M (2017). Long-term follow-up results of umbilical hernia repair. Wideochir Inne Tech Maloinwazyjne.

[13] Yao JJ, Pham T, El Mokdad A, Huerta S (2016). Predictors of recurrence of umbilical hernias following primary tissue repair in obese veterans. Am J Surg.

[14] Köhler G, Luketina RR, Emmanuel K (2015). Sutured repair of primary small umbilical and epigastric hernias: concomitant rectus diastasis is a significant risk factor for recurrence. World J Surg.

[15] Shankaran V, Weber DJ, Reed RL 2nd, Luchette FA (2011). A review of available prosthetics for ventral hernia repair. Ann Surg.

